# A constituent-based preprocessing approach for characterising cartilage using NIR absorbance measurements

**DOI:** 10.1088/2057-1976/2/1/017002

**Published:** 2016-01-18

**Authors:** Cameron P Brown, Minsi Chen

**Affiliations:** 1 Botnar Research Centre, NDORMS, University of Oxford, Old Road, Oxford OX3 7LD, UK; 2 Department of Computing and Mathematics, University of Derby, Kedleston Road, Derby DE22 1GB, UK; cameron.brown@ndorms.ox.ac.uk

**Keywords:** near infrared spectroscopy, cartilage, osteoarthritis

## Abstract

Near-infrared spectroscopy is a widely adopted technique for characterising biological tissues. The high dimensionality of spectral data, however, presents a major challenge for analysis. Here, we present a second-derivative Beer’s law-based technique aimed at projecting spectral data onto a lower dimension feature space characterised by the constituents of the target tissue type. This is intended as a preprocessing step to provide a physically-based, low dimensionality input to predictive models. Testing the proposed technique on an experimental set of 145 bovine cartilage samples before and after enzymatic degradation, produced a clear visual separation between the normal and degraded groups. Reduced proteoglycan and collagen concentrations, and increased water concentrations were predicted by simple linear fitting following degradation (all $p\ll 0.05$). Classification accuracy using the Mahalanobis distance was $\gt 98\%$ between these groups.

## 1. Introduction

Osteoarthritis (OA) is a degenerative joint disease that carries substantial personal and societal burden in its later stages. The development of OA involves a changes across the whole joint, with the loss of structural integrity of articular cartilage a central factor in the early disease process. Damage or degeneration detectable by visible light in common diagnostic procedures such as arthroscopy, however, are generally indicative of more severe structural degradation. Thus, detection of early stage degradation in articular cartilage is of special clinical interest to allow intervention before the disease burden manifests.

Spectroscopy-based techniques have been widely applied to characterise biological tissues, with Raman and mid-infrared spectroscopy commonly used [1]. Near infrared (NIR) spectroscopy, which measures harmonics and combinations of the fundamental molecular vibrations in the infrared energy range, is gaining popularity [2–6]. NIR compares favourably with other techniques for *in vivo* application [7], and has strong potential as a clinical tool. As NIR spectroscopy works with standard xenon light sources and fibreoptics, measurements can be taken with minimal instrument modification and without extra radiation delivered to the patient.

A major restriction to the use of NIRS, however, is the difficulty in interpretation in tissues due to the wide, overlapping spectral bands of the main extracellular matrix components, and thus a lack of independent peaks from which to base analysis. The high dimensionality of spectral data poses a further challenge to avoid overfitting, and requires very high sample numbers for robust training. A common and plausible solution is to pre-select optimal subsets of the spectrum for training. Selection methods based on wavelet coefficient regression and a genetic algorithm [8], mutual information and B-spline compression [9, 10], and mutual information and a modified genetic algorithm [11] have been successfully implemented.

Here, we take a relatively simple and physically based approach, using a Beer’s Law dimensionality reduction method to resolve spectral measurements into chemical concentrations. Intended as an input to more sophisticated predictive tools, this was coupled with linear least squares fitting, combining the second derivative spectra of matrix components to estimate their relative concentrations in the tissue. We tested our method’s capability to probe matrix composition in articular cartilage, first using a sample set of normal and enzymatically degraded bovine cartilage-on-bone.

## 2. Methods

### 2.1. NIR spectroscopy experiment

Absorbance spectra from 145 sample points on 6 macroscopically normal bovine patellae samples were collected using a Nicolet FT-NIR system (ThermoNicolet, UK) with a standard Michelson interferometer light path. The 4 mm diameter fibre optic probe was coupled to the FT-NIR system via a Grasby SPECAC NIR fibre port accessory (SPECAC, Orpington, UK), offset from the surface by ≈1.5 mm, based on the maximum signal from a diffuse reflectance standard. The spectrum was acquired from 4000 to 12 500 cm^−1^, with each measurement averaged over 124 scans ($\approx 40$ s). Patellae were immersed in 0.15 M saline between measurements. Samples were then enzymatically digested in 0.1 mg ml^−1^ of trypsin (from bovine pancreas T4665, Sigma–Aldrich, Australia) dissolved in 0.15 M phosphate buffered saline for 4 hours at 37 °C and retested. Removal of proteoglycans in the trypsin-treated samples was confirmed by safranin-O staining [12].

Spectra from isolated matrix components were collected using a Nicolet iS50 FT-NIR system with a standard SabIR fibre optic probe (Thermo Fisher Scientific, UK), offset from the surface by ≈4 mm, again based on maximum signal. Spectra of collagen (C9879, Sigma Aldrich, UK), chondroitin sulphate (C9819, Sigma Aldrich, UK) and distilled water were recorded and averaged over 64 scans ($\approx 10$ s). Cartilage and component spectra were taken in different laboratories, thus necessitating the use of different spectrometers. All spectra were reduced to the 4000–10 000 cm^−1^ (1–2.5 *μ*m) region, with 779 data points.

### 2.2. Data pre-processing

#### Data smoothing and derivation

The discrete absorbance measurements were first fitted with a cubic B-spline as discussed in [13]. The number of knots and their initial placement were determined by the uniqueness of each data point, with two consecutive data points *x*_*i*_ and ${x}_{i+1}$ considered unique if their difference is within a specified tolerance *τ*, i.e.$\ | {x}_{i+1}-{x}_{i}\parallel \leqslant \tau $. An empirically obtained noise model and an explicitly defined degree of freedom were used to preserve desired data characteristics. Equation (1) is the piecewise cubic B-spline fitting, *c*_*i*_ is a control point derived from the data points and ${B}_{i,3}$ is the *i*th cubic basis function

1 \begin{eqnarray*}A(\lambda )=\displaystyle \sum _{i=1}^{N}\;{c}_{i}{B}_{i,3}(\lambda ).\end{eqnarray*}

The use of piecewise fitting allows us to control the preservation of certain localised features in the data. Furthermore, since our subsequent dimensionality reduction method is dependent on the second derivative of the spectral data, this can be more conveniently achieved by taking the desired derivative of the basis function (see equation (2))

2 \begin{eqnarray*}\displaystyle \frac{{{\rm{d}}}^{2}A}{{\rm{d}}{\lambda }^{2}}=\displaystyle \sum _{i=1}^{N}\;{c}_{i}{B}_{i,1}(\lambda ).\end{eqnarray*}

#### Dimensionality reduction

One of the most challenging aspects of performing classification on spectroscopic data is its high dimensionality. The NIR spectrum covers the range between 800 and 2500 nm, and a typical input per spectrum is of the order of 1000 data points. The parameterisation of each sample over the NIR spectrum inevitably results in a large feature space. This also increases the number of training samples required for training a classifier.

Our approach aimed to reduce dimensionality by basing our analysis on the main constituents of the tissue: water, collagen and proteoglycan. Given the absorptivity of each individual component, the relationship between a mixture and its constituents can be described using Beer’s law. Equation (3) describes the specific formulation for modelling the absorbance of cartilage

3 \begin{eqnarray*}A({\lambda }_{i})={c}_{{\rm{w}}}l{\epsilon }_{{\rm{w}}}({\lambda }_{i})+{c}_{{\rm{c}}}l{\epsilon }_{{\rm{c}}}({\lambda }_{i})+{c}_{{\rm{p}}}l{\epsilon }_{{\rm{p}}}({\lambda }_{i})\end{eqnarray*}

$A({\lambda }_{i})$ is the absorptivity at wavelength ${\lambda }_{i};$ ${\epsilon }_{{\rm{w}}}$, ${\epsilon }_{{\rm{c}}}$ and ${\epsilon }_{{\rm{p}}}$ are the absorptivity of water, collagen and proteoglycan respectively; *c* is the concentration of each corresponding component, i.e. *c*_w_ for water, *c*_c_ for collagen and *c*_p_ for proteoglycan; *l* is the path length.The second order derivative of *A* is:

4 \begin{eqnarray*}\displaystyle \frac{{{\rm{d}}}^{2}A}{{\rm{d}}{\lambda }^{2}}={c}_{{\rm{w}}}l\displaystyle \frac{{{\rm{d}}}^{2}{\epsilon }_{{\rm{w}}}}{{\rm{d}}{\lambda }^{2}}+{c}_{{\rm{c}}}l\displaystyle \frac{{{\rm{d}}}^{2}{\epsilon }_{{\rm{c}}}}{{\rm{d}}{\lambda }^{2}}+{c}_{{\rm{p}}}l\displaystyle \frac{{{\rm{d}}}^{2}{\epsilon }_{{\rm{p}}}}{{\rm{d}}{\lambda }^{2}}.\end{eqnarray*}

If the absorbance of a mixture and the absorptivity its constituents are known across the NIR spectrum, the concentration of each component can be calculated by solving the following linear system:

5 \begin{eqnarray*}[\begin{array}{rcl}\displaystyle \frac{{{\rm{d}}}^{2}{\epsilon }_{{\rm{w}}}}{{\lambda }_{0}^{2}} &amp; \displaystyle \frac{{{\rm{d}}}^{2}{\epsilon }_{{\rm{c}}}}{{\lambda }_{0}^{2}} &amp; \displaystyle \frac{{{\rm{d}}}^{2}{\epsilon }_{{\rm{p}}}}{{\lambda }_{0}^{2}}\\ \displaystyle \frac{{{\rm{d}}}^{2}{\epsilon }_{{\rm{w}}}}{{\lambda }_{1}^{2}} &amp; \displaystyle \frac{{{\rm{d}}}^{2}{\epsilon }_{{\rm{c}}}}{{\lambda }_{1}^{2}} &amp; \displaystyle \frac{{{\rm{d}}}^{2}{\epsilon }_{{\rm{p}}}}{{\lambda }_{1}^{2}}\\ &amp; \vdots &amp; \\ \displaystyle \frac{{{\rm{d}}}^{2}{\epsilon }_{{\rm{w}}}}{{\lambda }_{k-1}^{2}} &amp; \displaystyle \frac{{{\rm{d}}}^{2}{\epsilon }_{{\rm{c}}}}{{\lambda }_{k-1}^{2}} &amp; \displaystyle \frac{{{\rm{d}}}^{2}{\epsilon }_{{\rm{p}}}}{{\lambda }_{k-1}^{2}}\end{array}][\begin{array}{c}{{c}_{{\rm{w}}}}^{l}\\ {{c}_{{\rm{c}}}}^{l}\\ {{c}_{{\rm{p}}}}^{l}\end{array}]=[\begin{array}{c}\displaystyle \frac{{{\rm{d}}}^{2}A}{{\lambda }_{0}^{2}}\\ \displaystyle \frac{{{\rm{d}}}^{2}A}{{\lambda }_{1}^{2}}\\ \vdots \\ \displaystyle \frac{{{\rm{d}}}^{2}A}{{\lambda }_{k-1}^{2}}\end{array}].\end{eqnarray*}

The least-square solution of the overdetermined linear system in equation (5) was obtained using QR factorisation.

Our use of Beer’s law omitted the path length (i.e. *l* = 1.0) because the variance of our bovine cartilage thickness is small (1.66 ± 0.25 mm) and thickness information is unavailable in proposed arthroscopic applications of the technique. This does imply that the solution for the component concentration levels is subjected to scaling. However, we demonstrate in the result section that this scaling does not affect the accuracy of our classification method.

By estimating the concentration of individual components using the aforementioned approach, the spectral data can be mapped into a lower dimensional feature space consisting of the primary constituents. In the case of cartilage, the salient components water, collagen and proteoglycan allow each sample to be parameterised by a feature vector $\vec{c}=({c}_{{\rm{w}}},{c}_{{\rm{c}}},{c}_{{\rm{p}}})$.

### 2.3. Classification

Based on results from unsupervised linear fitting described above, classification into normal and enzymatically degraded groups was performed based on the Mahalanobis distance. For a sample *x*, this was calculated as ${{\bf{D}}}^{2}={({\bf{x}}-{\boldsymbol{\mu }})}^{T}{{\bf{S}}}^{-1}({\bf{x}}-{\boldsymbol{\mu }})$, where *μ* is the mean of each group and ${\bf{S}}$ is the covariance matrix. Samples were classed as belonging to the training set to which the Mahalanobis distance was shortest.

The data pre-procssing and classification methods described here were implemented in R 3.1.2 (https://www.r-project.org).

## 3. Results and discussion

**Figure 1.**
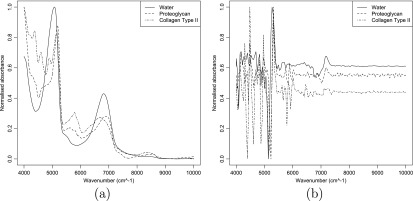
The NIR spectral data for the three primary constituents of articular cartilage: (a) absorbance; (b) second derivative.

**Figure 2.**
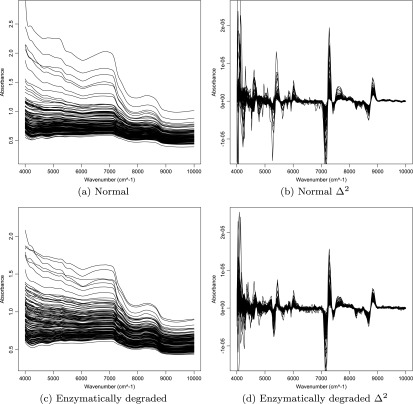
The NIR absorbance of articular cartilage from bovine knee joints fitted using cubic spline: (a), (b) the original and second derivative absorbance from normal samples; (c), (d) the original and second derivative absorbance from enzymatically degraded samples.

Spectra of the matrix components (water, proteoglycan and collagen) are presented in figure 1. Spectra of cartilage-on-bone samples before and after enzymatic digestion are provided in figure 2. Raw spectra of normal and trypsin-treated samples are offset, however this offset is likely to include systematic experimental variations between measurements. To circumvent this effect, second-derivative spectra were used due to intrinsic normalisation as well as the improved separation of spectral peaks. Even with second-derivative spectra, the lack of peak independence can be observed in figure 2, despite the clear differences in the isolated component spectra, thus prompting the mixture approach. The reader is referred to [4] for a description of peak assignments.

Unsupervised linear fitting of the three component spectra to sample spectra using equation (4) produced a clear visual separation of normal from enzymatically degraded samples. Using the Mahalanobis distance, normal and enzymatically degraded groups were classified with one false negative (0.34%) and four false positives (1.4%).

To examine the influence of omitting the thickness of individual sample, a path length randomly drawn from a normal distribution $(\mu =1.66,\sigma =0.25)$ was assigned to each cartilage sample. This is equivalent to applying an arbitrary scaling to the solution of equation (5). This test was repeated *N* = 100 times where *N* was chosen to be 2/3 of the sample size in each cluster. Table 1 shows that a small variance can be observed from the centre of each cluster in the feature space when path lengths were factored in. However, this variance has a negligible impact on the stability of our classification method as shown in table 2. The mode of false positive and false negative count remained identical to the results where the path length was postulated to be one. It should be noted that degradation by trypsin has a negligible effect on sample thickness [14].

**Table 1.** The influence of scaling on the mean of each cluster *N* = 100.

**Cluster**	**Mean *c*_w_**	**Mean *c*_c_**	**Mean c_p_**
Normal	0.1418 ± 0.0169	0.5949 ± 0.0446	0.5571 ± 0.0517
Degraded	0.4052 ± 0.0346	0.2486 ± 0.0316	0.2237 ± 0.0237

**Table 2.** The influence of scaling factor on the stability of classification performance; the worst case for false positive is $2.1\%$ and for false negative is $1.7\%$ *N* = 100.

**classification**	**Mode**	**Min**	**Max**
False pos. # (%)	4 $(1.4\%)$	3 $(1.1\%)$	6 $(2.1\%)$
False neg. # (%)	1 $(0.34\%)$	0 $(0\%)$	5 $(1.7\%)$

**Figure 3.**
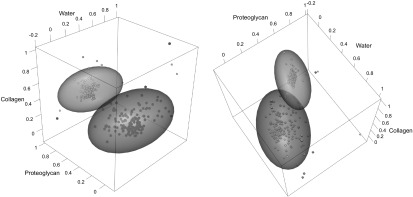
Predictions from unsupervised fitting of water, proteoglycan and collagen contributions to tissue spectra. Ellipses show the covariance matrices of the respective samples, used for calculation of Mahalanobis distance. The green spheres and ellipse represent normal samples; red cubes and ellipse represent enzymatically degraded samples.

Visual trends (figure 3) for proteoglycan and water predictions conformed to expectations: enzymatic degradation reduced predicted proteoglycan content and increased predicted water content (both $p\to 0$), filling the space left by proteoglycan removal. Predicted proteoglycan and water content was negatively correlated (*r* = −0.74) for pooled sample groups. Predicted collagen content also decreased after enzymatic degradation ($p\to 0$). We interpret this as a secondary effect of trypsin on collagen by either restructuring in the absence of proteoglycan affecting scattering or cleavage reducing collagen content.

It should be noted that study is limited by a lack of ground truth measurements of constituent concentrations in each sample. Safranin-O staining confirmed loss of proteoglycan content following enzymatic degradation, however this was not quantified and pre-degradation staining was not possible. Solution stability in least squares fitting of bovine samples was confirmed by subsampling with spectral windows. Negligible fluctuation was observed in estimated concentrations, however, the optimisation and minimisation of spectral range for prediction was not explored here. Spectral optimisation and comparison of predictions with ground truth biochemical data will be undertaken in future work.

Applying Beer’s law to second derivative spectra, we have developed a preprocessing technique with a physical basis for dimensionality reduction to estimate concentrations of tissue matrix components from spectroscopy. Demonstrating its application to NIR spectroscopy of cartilage, we found that simple linear fitting of isolated second derivative spectra of water, proteoglycan and collagen could be used to separate normal from enzymatically degraded cartilage. We propose that this approach will provide a physically-based, low dimensionality input into more advanced classification strategies [15] such as an artificial neural network to predict absolute concentrations of matrix components. This approach may further be used in a range of soft tissues, such as cornea, skin, inter vertebral disk and tendon, in which the main matrix components are near infrared-active.
